# Elastic Anisotropy in BCC Ti-X Alloys (X = V, Nb, Ta) Determined from First Principles

**DOI:** 10.3390/ma18184294

**Published:** 2025-09-12

**Authors:** Cyprian Sobczak, Piotr Kwasniak, Pawel Strak, Marek Muzyk, Stanislaw Krukowski

**Affiliations:** 1 Institute of High Pressure Physics, Polish Academy of Sciences, Sokolowska 29/37, 01-142 Warsaw, Poland; csobczak@unipress.waw.pl; 2 Multidisciplinary Research Center, Cardinal Stefan Wyszynski University in Warsaw, Dewajtis 5, 01-815 Warsaw, Polandm.muzyk@uksw.edu.pl (M.M.)

**Keywords:** titanium alloys, ab initio modelling, elastic anisotropy

## Abstract

Elastic isotropy is a phenomenon in which a material responds uniformly to stress, regardless of its direction. In the case of cubic crystals, which possess distinct crystallographic directions, this represents a remarkable manifestation of quantum mechanics in macroscopic objects. Such behavior of a crystal cannot be explained within the framework of classical physics. The phenomenon is closely related to the balancing of internal forces resulting from Coulomb interactions, Pauli repulsion, and the overlap in the bands when stress is applied to the crystal. On the macroscopic level, this corresponds to the relationship between elastic constants given by 2 C_44_/(C_11_ − C_12_) = 1. The subject of the present work is to demonstrate the influence of the number of valence electrons per atom in binary titanium alloys with vanadium, niobium, and tantalum on the shape of the anisotropy curve. The result of the work is the identification of a new Ti-53Nb alloy exhibiting elastic isotropy, and the demonstration that this phenomenon cannot occur for TiTa alloys, in the range of mechanical stability of these alloys. This study includes a summary of the main trends exhibited by the elastic constants, Young’s modulus, and bulk modulus of the discussed Ti-based alloys, based on ab initio methods. Additionally, the work addresses the well-known difficulty in determining the elastic constants of vanadium and niobium, along with a proposed solution that offers significant improvement in reproducing experimental results compared to the conventional use of the PBE (Perdew–Burke–Ernzerhof) functional.

## 1. Introduction

Elastic isotropy is a property of a material manifested by an identical response to applied stress, regardless of the crystallographic direction. Technically, this means that both the Young’s modulus *E* and the shear modulus *G* are the same for all crystallographic directions [1,2]. Such a unique property of materials is a kind of anomaly in crystalline structures—it occurs, among others, in tungsten and in the Ti-72V alloy [3].

Crystals with elastically isotropic properties distribute stress equally to neighboring grains during force application, which makes the material more resistant to fatigue. This enables increased durability, which is particularly important in medical (implants), structural, and aerospace applications [4,5]. Elastic isotropy gives extraordinary strength to polycrystalline metal material against cracking under thermal and mechanical stress. Despite the obvious advantages, elastic isotropy in metals remains a relatively poorly studied feature with the exception of the Tian et al. work [6]. It cannot be explained solely by electrostatic mechanisms, and in addition, it has deep quantum foundations.

The mechanical properties of a material are influenced by the following components: *W_e_*—electrostatic interactions between ions and electrons and *W_r_*—repulsions resulting from the overlap of electronic bands originating from individual ions [7,8]. The final and probably dominant contribution comes from subtle electronic effects related to the value of the Fermi energy and the shape of the Fermi surface. This component, denoted as *W_f_*, has a significant influence on the shear modulus, and thus on the elastic constant *C*_44_ (in cubic structures), and therefore clearly depends on the number of electrons in the *d*-shell. A strong correlation has been observed between the *e/a* ratio (the number of electrons per atom) in transition metal alloys and the Zener anisotropy factor *A* [3,9]. Zener initiated considerations on the anisotropy of cubic crystals by introducing classification criteria for materials. Based on the mechanical stability condition (*C′* > 0, where *C′* = (*C*_11_ − *C*_12_)/2), he observed that for *A* = *C*_44_/*C′*, the material exhibits elastic isotropy. Zener [9] emphasized the important role of the relationship between the response of the second and nearest neighbors in the BCC structure to changes in interatomic distance, highlighting its significance in the phenomenon of elastic isotropy (he suggested that second neighbors respond more strongly to distance changes). It is currently believed that elastic isotropy in crystals occurs when the contributions *W_e_*, *W_r_*, and *W_f_* mutually compensate one another. The relation of the C’ elastic modulus to stability of BCC transition metals was the subject of the study of Fisher et al. [10].

Proceeding from extremal principles of elasticity, Ranganathan et al. [11] introduced a new universal anisotropy index. Furthermore, they established special relationships between the proposed anisotropy index and the existing anisotropy measures for special cases. A new elastic anisotropy diagram was constructed for over 100 different crystals (from cubic through triclinic), demonstrating that the proposed anisotropy measure is applicable to all types of elastic single crystals, and thus fills an important void in the existing literature. Another example of definition of an anisotropic factor was proposed by Kube [12].

The importance of the Fermi surface shape for the physical properties of metals was already considered earlier in the context of Van Hove singularities [13] and the associated Lifshitz topological transitions [14]. This phenomenon is, in essence, the deformation of the Fermi surface, resulting in its constriction. The appearance of the so-called “neck” results in a sudden change in physical properties, such as electrical resistivity or a drop in the values of elastic constants. Changes in these physical quantities can, to some extent, be controlled by influencing the Fermi level via pressure, magnetic field, or the number of electrons in the conduction band [15,16,17,18,19]. It is assumed that the unusual shape of the Fermi surface is the source for the change of the characteristic distribution of the *A*(*e/a*) function (where e/a is weakly bound electrons from the outer shells per atom), which takes a parabolic form, with a visible minimum of the anisotropy factor [3,20,21].

Although the condition for elastic isotropy is quite clear, it presents many challenges in the design of such alloys. Ab initio methods are very well suited to reproducing certain general trends exhibited by materials depending on the chemical composition. However, when predicting specific material properties, difficulties arise due to the influence of phonons or the temperature dependence of elastic constants. In comparisons with experimental data, contamination by unwanted elements is inevitable, which also affects the relationship between theory and measurement. In theoretical considerations of elastic isotropy in transition metals, a particularly important issue turns out to be the precise determination of the elastic constant *C*_44_. In some elements, such as Mo, Ta, or W, this problem does not manifest significantly, or it can be controlled by an appropriate set of parameters that allows good agreement with experimental results. In the case of metals such as V and Nb, however, this difficulty becomes severe, leading to a reduction of the anisotropy factor by almost half. The problem of drastically underestimated *C*_44_ in V and Nb has been known for many years; researchers attribute its cause to the overly localized metallic bonding within the PBE framework [22,23,24].

In this article, we provide an ab initio modelling of titanium containing alloys with certain metals. We relate the anisotropy factor with respect to the number of valence electrons per atom (e/a) and also elastic constants, bulk modulus, and Young modulus. The main finding is that we found a new alloy, i.e., Ti-53Nb, which is isotropic. We hope that the theoretical work presented in this paper will inspire experimentalists to obtain this new alloy, which then could lead to practical applications.

## 2. Methodology

### 2.1. Computational Setup

This study was conducted within the framework of density functional theory (DFT), implemented in the VASP computational package [25,26,27,28], which utilizes the Projector Augmented-Wave method (PAW) [29]. The applied functional approximation was based on the generalized gradient approximation (GGA), using the PBE functional [30]. In the case of corrections for vanadium and niobium (the RP-Revised PBE from Hammer et al. [31]) and ML (PW86R exchange [32] + PBE correlation [30]) functionals were used, which will be discussed in more detail when addressing those specific cases.

For titanium, vanadium, and tantalum, *pv*-type PBE pseudopotentials were applied, allowing for semi-core *p*-electrons, with a plane-wave cutoff energy of 500 eV. Niobium required the use of *sv*-type pseudopotentials (PBE), which include deeper *s*-electrons, and a plane-wave cutoff energy of 600 eV. Atomic models consisting of 128 atoms (4 × 4 × 4 supercell, with periodic boundary conditions) were generated using the ATAT program via the SQS (special quasi-random structure) method [33]. The structure is created based on a statistical Monte Carlo method and the measurement of correlations between neighboring atoms, aiming to achieve maximum disorder [33]. The *k*-point sampling, in the case of alloys with niobium and tantalum, required a 4 × 4 × 4 mesh, whereas for alloys with vanadium (RP), a 3 × 3 × 3 mesh was used with a cutoff energy of 600 eV. While testing the nonstandard ML functional for the Ti–Nb alloy variant, it also required adjusting the plane-wave cutoff to 550 eV due to high RAM (random access memory) usage. The ground state of each structure was determined based on both volume and ionic relaxation, using the Hellmann–Feynman force convergence criterion. The relaxation was stopped when the difference in the forces acting on all atoms were less than 0.02 eV/Å. The SCF (self-consistent field) loop was stopped when energies of the two consecutive iterations were less than 2.5 × 10^−6^ eV. For alloys containing vanadium and niobium, volume and ionic relaxation were performed separately due to the stability of the systems within the considered composition range, which yielded results consistent with single-step relaxation. In the case of alloys containing tantalum, at very low titanium concentrations, the cells tended to tilt slightly, but without significant deviation from the BCC structure. Ti-Ta alloys required simultaneous volume and ionic relaxation to obtain a state free of internal stresses.

To calibrate the parameters for Ti, a brief test was performed on a single HCP (Hexagonal Close-Packed) unit cell using the pv pseudopotential, a 16 × 16 × 10 k-point mesh with 500 eV plane-wave cutoff, and the remaining settings as specified above.

### 2.2. Determination of Elastic Constants

By determining the ground state of a crystal structure, we obtain information about its total internal energy (excluding phonon contributions). If the considered structure is then subjected to a small, permanent deformation and subsequently relaxed again, we can determine the energy required to induce such a deformation. In other words, we can calculate the difference between the energy of the ground state and the deformed state and relate it to the well-known Taylor series expansion of Hooke’s law [34,35,36]:

(1) $$E\left(V,\varepsilon \right)=E_{0}+V_{0}\left(\sigma_{ij}\varepsilon_{ij}+\frac{1}{2}C_{ijkl}\varepsilon_{ij}\varepsilon^{kl}\right)$$

where σ_ij_ is the stress tensor, ε_ij_ is the strain tensor, E_0_ is the ground state energy (without deformation), and V_o_ is the volume of the undeformed structure. C_ijkl_ is the stiffness tensor, which, in the case of a BCC structure, reduces to only three independent constants, expressed in Voigt notation as C_11_, C_12_, and C_44_. The middle term in Equation (1) is neglected due to its large contribution from the first derivative with respect to strain. In this method, attention is focused on the quadratic term, which contains the factor representing the relationship with the elastic constants. Therefore, by properly selecting the deformation, the values of the elastic constants can be obtained directly from the energy. Deformation modeling is carried out by multiplying the edges of the cubic unit cell by appropriate deformation matrices (Equations (2)–(4)). The matrix generated according to this scheme forms the basis for the structure, already including the desired deformation. (Equations (2)–(4) constitute not only one valid set of deformation matrices) [35,36,37,38].

(2) $$D_{I}=\left[\begin{array}{ccc} 1+\varepsilon & 0 & 0\\ 0 & 1-\varepsilon & 0\\ 0 & 0 & 1/\left(1-\varepsilon^{2}\right) \end{array}\right]$$

(3) $$D_{II}=\left[\begin{array}{ccc} 1+\varepsilon & 0 & 0\\ 0 & 1+\varepsilon & 0\\ 0 & 0 & 1+\varepsilon \end{array}\right]$$

(4) $$D_{III}=\left[\begin{array}{ccc} 1 & \varepsilon /2 & \varepsilon /2\\ \varepsilon /2 & 1 & \varepsilon /2\\ \varepsilon /2 & \varepsilon /2 & 1 \end{array}\right]$$

The strain values e adopted for all considered alloys are −0.015, −0.01, −0.005, 0.005, 0.01, and 0.015. For such prepared deformations, the energy expansions presented in Equations (5)–(7) are applied, neglecting higher-order terms.

(5) $$E_{I}\left(V,\varepsilon \right)=E_{0}+V_{0}\left[\left(C_{11}-C_{12}\right)\varepsilon^{2}+O{(\varepsilon }^{4})\right]$$

(6) $$E_{II}\left(V,\varepsilon \right)=E_{0}+V_{0}\varepsilon \left(\sigma_{1}+\sigma_{2}+\sigma_{3}\right)+V_{0}\left[\frac{3}{2}\left(C_{11}+2C_{12}\right)\varepsilon^{2}+O(\varepsilon^{4})\right]$$

(7) $$E_{III}\left(V,\varepsilon \right)=E_{0}+V_{0}\left[\frac{3}{2}C_{44}\varepsilon^{2}+O(\varepsilon^{4})\right]$$

Based on the matrices and Equations (2)–(7), three sets of energy versus deformation functions are constructed, each exhibiting a parabolic distribution. The slope coefficients are represented by the elastic constants, multiplied by appropriate factors depending on the applied deformations. After determining the constants, each structure is rotated, and the procedure is repeated using different model orientation. Such approach minimizes an influence of possible atomic segregation on the evaluation of mechanical properties. Based on the thus-determined elastic constants, the bulk modulus *B* and Young’s modulus *E* are calculated using the well-known Equations (8) and (9) [35].

(8) $$B=\frac{1}{3}\left({\bar{C}}_{11}+2{\bar{C}}_{12}\right)$$

(9) $$E=\frac{1}{2}\left[\frac{9}{1/B+15/\left({\bar{C}}_{11}-{\bar{C}}_{12}+3{\bar{C}}_{44}\right)}+\frac{5}{5/9B+4/3\left({\bar{C}}_{11}-{\bar{C}}_{12}\right)+1/{\bar{C}}_{44}}\right]$$

where the symbols C denote the average values of the elastic constants obtained from each of the three spatial orientations of the model.

## 3. Results and Discussion

The previously mentioned issues related to performing calculations using the PBE functional for pure V and Nb cannot be resolved by appropriately adjusting the parameters. As a measure of last resort, the possibility of forcing the use of other functionals in the VASP computational package functionals incompatible with the PBE pseudopotentials was employed. The results of this workaround turned out to be surprisingly close to the experimental data. Due to the fact that such a method of performing calculations may raise concerns, the anisotropy curves for the TiV and TiNb alloys were also determined using the conventional method with PBE. Table 1 presents a summary of the results for the elastic constants, elastic moduli, and the anisotropy factor for the pure elements.

Titanium in its pure form does not adopt the BCC structure below 882 °C. Consequently, attempts to model the elastic constants of BCC Ti using standard DFT (which assumes 0 K) are unsuccessful. Such attempts yield results E < 0 and A < 0, yet we can find these values in the literature [40,41]. An additional complication is the lack of experimental elastic-constant data for BCC Ti. For this reason, we selected to test parameters on a single HCP unit cell—the stable structure of Ti below 882 °C—and to compare the resulting elastic constants with experimental data. The results summarized in Table 2 indicate a close similarity between the findings of this study, Chihi et al. ab inito modelling [42] and the experimental data for HCP Ti [43].

### 3.1. TiV Alloys

**Figure 1:**
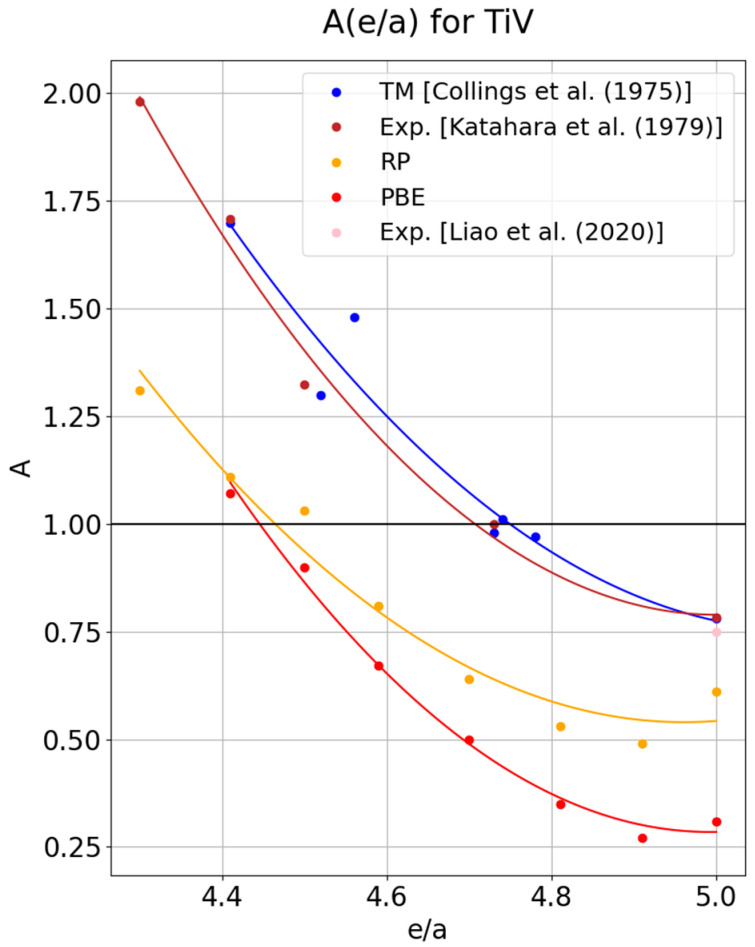
Relation of the anisotropy factor with respect to the number of valence electrons per atom (*e/a*), determined experimentally for transition metals (blue line) [3], and experimental results for vanadium (pink point) [41], as well as for TiV alloys determined experimentally (brown line) [44], using the RP functional (orange line), and using PBE (red line).

As shown in Figure 1, the plot presenting the results for TiV alloys can be reasonably well approximated by a second-degree polynomial. The points obtained using PBE (red curve) show very little scatter around the curve; a slightly larger deviation is observed for the curve calculated with the RP functional [38] (orange curve), which, on the other hand, reproduces the properties of pure vanadium much better. In the case of the RP method, the anisotropy factor for pure vanadium is 0.61, whereas for PBE it is 0.3. In reference [18], using molecular dynamics methods, researchers achieved significantly better results than those from PBE, yet still far from the experimental values, in which the anisotropy factor is 0.78. The brown points represent the results of experimental measurements for TiV alloys. The blue line is an approximation of experimental results, but it was not derived from any specific alloy; instead, it refers to various transition metal alloys, including the elastically isotropic Ti-72V alloy. The PBE line captures the slope of the distribution well; however, despite the interesting fact that the PBE line almost intersects the RP line exactly at the point corresponding to elastic isotropy for *e/a* = 4.45, this is not the *e/a* value at which the phenomenon is actually observed in experiments. What should be emphasized is that the authors of [41] obtained an accurate result for an elastically isotropic alloy using ab initio methods; however, they employed the virtual crystal approximation (VCA) to generate structures, rather than SQS as in this work (A = 1, for Ti-73V). Despite the precise agreement between the value predicted in [41] and the experimental results for the Ti–72V alloy, pure vanadium still lies beyond the method’s reach (according to [41], the anisotropy factor for vanadium is 0.37). Both methods (RP and PBE), however, confirm the existence of a point with elastically isotropic properties. Both curves determined in the present work allow for observation of the parabolic character of the distribution, resulting from the minimum of A occurring at e/a = 4.91.

### 3.2. TiNb Alloys

**Figure 2:**
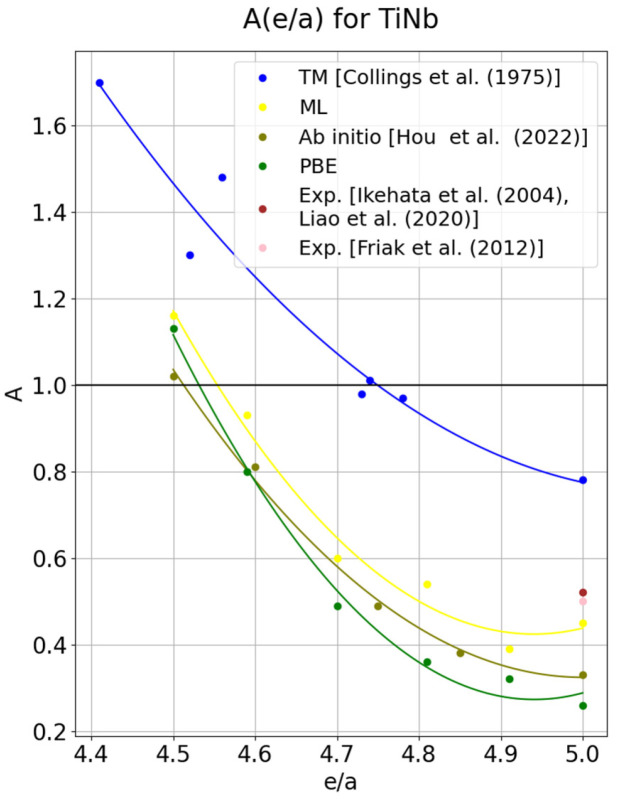
Relation of the anisotropy factor with respect to the number of valence electrons per atom (*e/a*) determined experimentally for transition metals (blue curve) [3], and for TiNb alloys determined using the ML functional (yellow line), PBE (green curve), and ab initio by another author (olive line) [45]. The brown and pink point represents the experimentally determined value for pure Nb [39,41,50].

In the case of TiNb alloys, once again, a much better approximation for pure niobium is provided by the ML functional (yellow curve in Figure 2). It allows for obtaining an anisotropy factor of 0.45, while the result from PBE is *A* = 0.26 (green line), with the experimentally determined value being 0.52. For TiNb alloys, no experimental results are found in the literature; therefore, the results were compared with theoretical studies [45,46] (olive line). The results turned out to be very similar for lower *e/a* values, particularly in the region of expected elastic isotropy. According to reference [45], the value *A* = 1 is obtained near *e/a* = 4.51; for PBE in that study, it is *e/a* = 4.43, and using ML it is *e/a* = 4.45. All predictions confirm the existence of elastic isotropy. Analysis of the anisotropy curve for Ti–Nb, as well as for Ti–V, was also undertaken in [47], but the Ti–Nb alloys considered there lie in the e/a range of 4.2–4.4, whereas the present work examines the anisotropy behavior in the range e/a = 4.5–5.0. As emphasized in Refs. [20,23,24], properly determining the elastic constants for pure niobium is an extremely problematic task. For the ML functional calculations are time-consuming and numerically unstable; in several cases, they even required removal of some points on the strain parabola due to large deviations. Nevertheless, the good approximation of results on this functional for niobium-based alloys is confirmed. For TiNb alloys, as in the case of TiV, the molecular dynamics methods were applied [46]. The results obtained in the present work are not consistent with those from Refs. [48,49]. However, they are close to the results reported in Ref. [40]. A reliable modeling of the behavior of titanium–niobium-based alloys can serve as a valuable direction in the development of these alloys in the context of shape memory alloys [48,49]. Figure 2 presents a comparison of lines obtained using PBE (green line) and ML (yellow line), as well as the line obtained in Ref. [45], that was also determined using ab initio methods. The illustration shows, as in the case of vanadium, the lines obtained for transition metals and the experimental point that were determined only for pure niobium. Results in Figure 2 prove that the minimum of the anisotropy curve for TiNb alloys is obtained when using the ML functional at *e/a* = 4.91. In the case of PBE, the regression curve also indicates the presence of a minimum, although it is not highlighted by a specific point.

Similar analyses of elastic isotropy were reported in [51], but over a composition (concentration) range that precludes a meaningful comparison (%at.: 10–45).

### 3.3. TiTa Alloys

**Figure 3:**
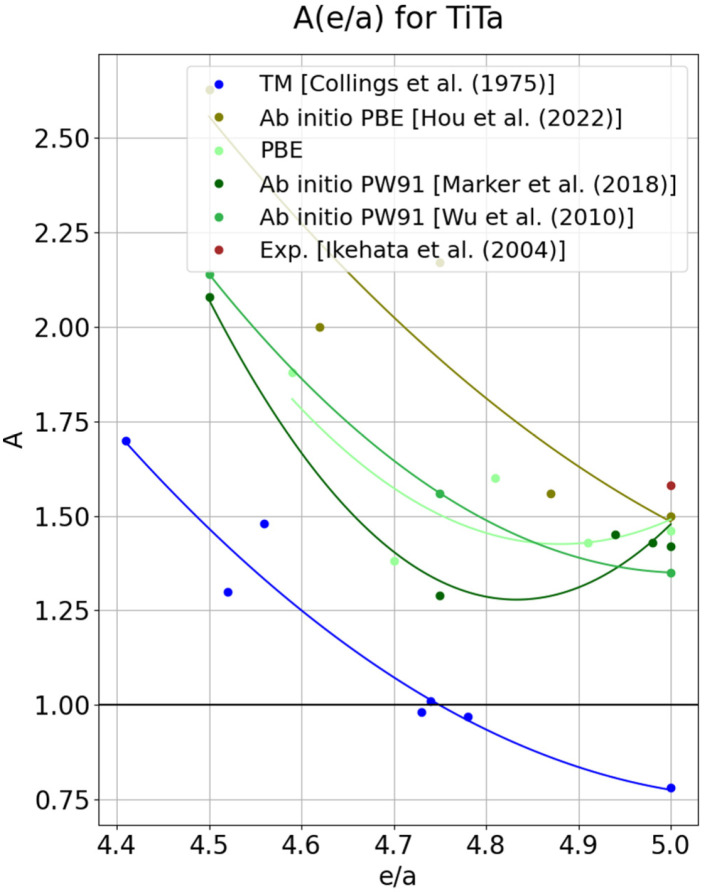
The anisotropy factor in function of the number of valence electrons per atom (*e/a*), determined experimentally for transition metals (blue points) [3] and for TiTa alloys calculated using the PBE functional (dark green [52], light green line [53]), and by ab initio calculations in Ref. [45] (olive line). The brown point represents the experimentally determined value for pure Ta [39].

The anisotropy curve for Ti–Ta alloys exhibits a very high scatter of data points around the regression curve. This issue is not limited to the curve obtained in the present work using PBE; it is also visible in other authors’ results (Refs. [45,52,53]). The curves shown in Figure 3 display a fairly similar character, decreasing gently with increasing e/a. The high degree of similarity among the anisotropy curves reported by other authors influenced the decision in this work to discard the point corresponding to e/a = 4.5, where the curve took the value A = 1.53. Including this point drastically affected the course of the curve, strongly flattening it. A likely reason for such a strong deviation from the trend line for the remaining points is the effect of atomic arrangement within the cell. In mixtures of elements such as Ti and V, the atomic distribution in the cell has a less drastic impact on the alloy’s mechanical properties. In the case of Ti and Ta, differences in atomic size may prove significant. Supporting this argument is the observation that for low Ti contents in Ti–Ta, all curves lie close to each other, which changes markedly as e/a decreases, i.e., as the Ti concentration increases. The size of the cell considered also appears to have a significant effect. In Refs. [45,53], the calculations were carried out for 16-atom cells, which, under periodic boundary conditions, inevitably impose a strong ordering of the lattice. The study in Ref. [52] used 54 atoms, whereas the present work uses 128.

Despite the use of the PW91 functional in Refs. [52,53], the anisotropy curves do not show particularly strong similarity. We therefore conclude that the size difference between Ti and Ta unexpectedly increases the dependence on the type of atomic mixing in the supercell model. There is, however, no doubt that within the e/a range considered here, no elastically isotropic alloy is observed; moreover, judging from the character of the curves, no binary Ti–Ta alloy exhibits elastic isotropy. Although two of the four curves appear to possess a minimum in the anisotropy, the analysis of the elastic constants in Figure 3 does not indicate anomalies that could be associated with a van Hove effect.

### 3.4. Comparison of Anisotropy Curves for Ti-X Alloys (X = V, Nb, Ta, Mo)

**Figure 4:**
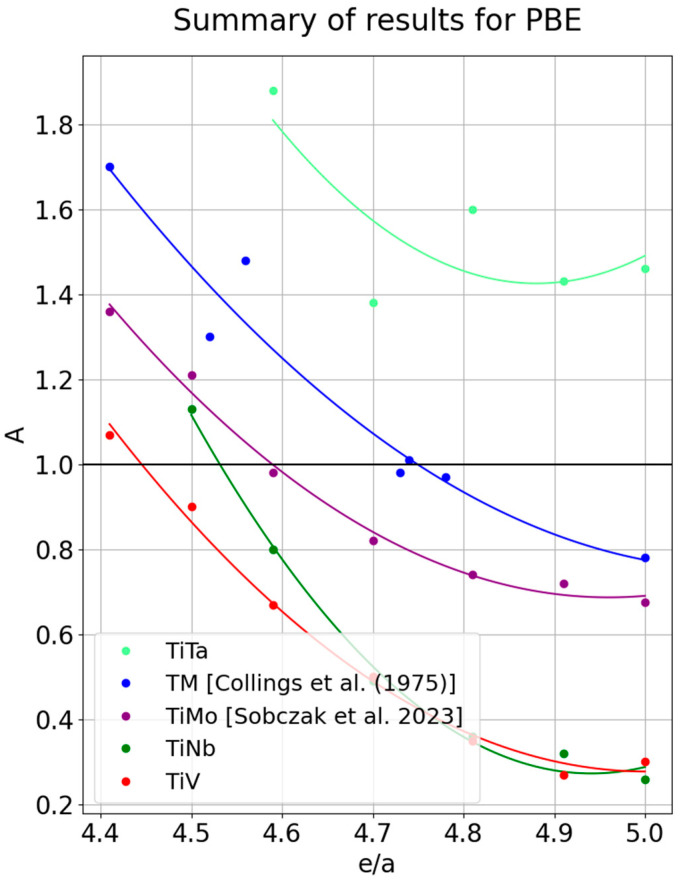
Comparison of anisotropy determined in this work for TiV alloys (red line); TiNb (green line); TiTa (light green line), and the results for TiMo alloy (violet line) obtained in Ref. [25]. The blue line represents the approximation for transition metals [3].

Figure 4 presents a comparison of all lines considered, in addition to the violet line obtained in Ref. [25], representing TiMo alloys investigated using the same method. It is plain to see that the presented lines differ among themselves, particularly in the case of TiNb alloys, which appear to intersect the lines for TiV and TiMo. The TiTa line exhibits a completely different character than the other ones, not reaching the value *A* = 1 at any point. It should therefore be assumed that the trends determined using PBE indicate more complex relationships for the anisotropy factor than those merely based on the number of electrons per atom. Otherwise, one would expect the possibility that these lines would be by a simple translation by a constant value.

Based on the presented results, the conjecture in Ref. [3]—that the dependence of A on e/a has a decisive influence on the distribution of points along the curve—can therefore be ruled out. This, in turn, shows that the attempt to describe all transition metal alloys by approximating them with a single anisotropy curve, as done in Ref. [3], is insufficient.

### 3.5. General Trends in Ti-X Alloys (X = V, Nb, Ta, Mo)

**Figure 5:**
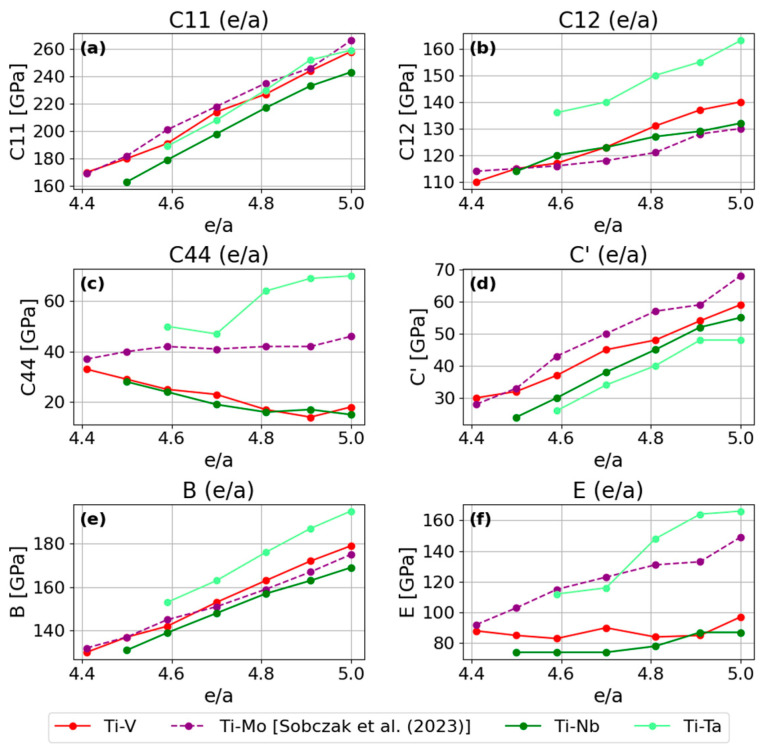
A comparison of trends observed in Ti-X alloys (X = V, Nb, Ta, Mo [25]): (**a**) *C*_11_ elastic constant, (**b**) *C*_12_ elastic constant, (**c**) *C*_44_ elastic constant, (**d**) *C′* = (*C*_11_ − *C*_12_)/2, (**e**) bulk modulus *B*, and (**f**) Young’s modulus *E*, determined using PBE.

The trends exhibited by titanium alloys with the considered transition metals as a function of the *e/a* ratio are presented in Figure 5. An increase of almost all elastic constants with the *e/a* ratio is noticeable.

An exception is provided by the Ti–V and Ti–Nb alloys, for which a decrease in the C44 constant with increasing e/a is evident. In the Ti–V alloy, a minimum of C44 is observed near e/a ≈ 4.91, after which it increases slightly at e/a = 5. In view of the side effects noted in the introduction on the van Hove effect—such as unexpected changes in mechanical properties—one may surmise that at e/a = 4.91 when the Fermi surface assumes an unusual shape. Although the difference in the behavior of C44 is visible, it is not dramatic.

For Ti–Nb, the C44(e/a) trend appears to reach its lowest point at pure niobium, without pronounced deviations. Based on the set of elastic constants, one cannot unambiguously identify a point that would suggest the presence of a van Hove effect known to occur in niobium under pressure. In the case of the Ti–Mo alloy, the C44(e/a) curve does not suggest any anomalous effects. The slope of the curve does change, but only very slightly. For Ti–Ta, although there is a point that differs from the others in the C44(e/a) profile, it appears quite early and therefore cannot be associated with the minimum in the anisotropy curve. Moreover, as noted in the section on Ti–Ta alloys, at high titanium contents (i.e., lower e/a) these alloys are more sensitive to the atomic mixing configuration, which manifests under shear deformation of the cell. For this reason, based on the elastic constants alone, one cannot conclude that an anomaly is present in Ti–Ta.

To establish whether a deformation of the Fermi surface is present, density-of-states (DOS) curves must be determined.

The bulk modulus B for all of the alloys considered shows the same increasing trend, with comparable linear slopes. The Young’s modulus E likewise increases for the Ti–Ta and Ti–Mo alloys. For Ti–V alloys, E falls in the range 83–97 [GPa], whereas for Ti–Nb alloys, it lies in the range 74–87 [GPa].

Table 3 compiles binary Ti alloys predicted to be elastically isotropic, determined theoretically and compared with experimental results where possible. The immediately apparent discrepancy—namely, the experiment indicating elastic isotropy for Ti–72V versus the markedly different prediction for Ti–45V—can be attributed to the fact that even approximating the anisotropy factor for pure vanadium carries substantial uncertainty. In the section devoted to Ti–V alloys, we explained the considerable effort made to approximate as accurately as possible the mechanical properties determined for V, which ultimately did not yield the desired outcome. The method employed in the present work also produces a correct result for W, for which A = 0.97, very close to the A = 1 value for which tungsten is well known. For the Ti–53Nb and Ti–30Mo [25] alloys, the theoretical predictions cannot be directly confronted with measurements. Nevertheless, the Ti–53Nb alloy is particularly promising and awaits experimental confirmation of its elastic isotropy.

## 4. Summary and Conclusions

One benefit of the present work was to highlight the relationship between the anisotropy factor and the ratio of weakly bound valence electrons per atom (e/a) in binary titanium alloys with transition metals. This relationship is evident—though it differs from alloy to alloy—which provides an important guideline for further studies on Ti–transition-metal alloys. Moreover, as a byproduct, we attempted to detect an anomaly in the C44 constant as a function of e/a, which could indicate a van Hove effect associated with a stress- or electron-density-induced deformation of the Fermi surface. Such an effect should manifest as an atypical drop in C44 in the alloy under study. This type of observation was made only for vanadium, where a slight decrease of C44 was recorded at e/a = 4.91. Further development of the discussion of anomalies related to the Fermi surface shape will require presenting density-of-states (DOS) curves, and a definitive resolution will demand appropriate experiments that map the Fermi surface.

The main goal of the work was to determine—or ascertain the existence of—compositions with elastically isotropic properties (A(e/a) = 1) in Ti–Nb and Ti–Ta alloys. We also performed tests intended to reproduce, theoretically, known isotropic cases such as Ti–72V and W. While with the present method, the agreement between prediction and experiment was confirmed for tungsten, the theoretical confirmation of the Ti–72V composition in Ti–V was not successful. The composition indicated by the theoretical approach was Ti–45V, which we attribute to the difficulty of correctly determining the anisotropy factor for pure vanadium. It is well known that PBE does not accurately capture all bonding effects in this element.

Using the method presented here, we established that an elastically isotropic Ti–Ta alloy does not exist. We also identified an elastically isotropic Ti–53Nb alloy. Because of the presence of unusual electronic effects in pure niobium (though not as pronounced as in vanadium), other functionals were also tested. The ML functional, which gives a good approximation of the anisotropy factor for pure Nb, when applied to Ti–Nb yielded nearly the same result, indicating an isotropic composition at e/a = 4.55. Obtaining nearly identical compositions with two different functionals supports the reliability of this prediction. To determine unambiguously whether Ti–53Nb is indeed elastically isotropic, experimental verification will be required.

A further research perspective in this field involves determining analogous curves for hafnium- and zirconium-based alloys with early transition metals, thereby providing a complete picture of the character of anisotropy curves. Advancing knowledge of this unusual phenomenon may, in the future, lead to the development of structural materials with greater fatigue resistance, enabling industry—and even the construction sector—to save substantial costs otherwise spent on renovation and maintenance.

## Figures and Tables

**Table 1:** The comparison of the results obtained in this work using the PBE functional, as well as RP for vanadium and ML for niobium, with experimental data and ab initio results reported by other researchers [39].

Metal	Source	Functional	B [GPa]	G [GPa]	E [GPa]	C_11_ [GPa]	C_12_ [GPa]	C_44_ [GPa]	A
V	This work	PBE	192	50	137	258	139	18	0.3
V	this work	RP	174	54	147	268	127	43	0.61
V	Ab initio [39]	PBE	183	41	114	270	140	25	0.38
V	Exp. [39]	-	156	48	130	229	119	43	0.78
Nb	This work	PBE	169	31	87	243	132	15	0.26
Nb	This work	ML	163	35	99	234	128	24	0.45
Nb	Ab initio [39]	PBE	172	32	91	247	134	16	0.28
Nb	Exp. [39]	-	171	40	111	246	134	29	0.52
Ta	This work	PBE	195	61	166	259	163	70	1.46
Ta	ab initio [39]	PBE	190	63	170	257	156	71	1.41
Ta	Exp. [39]	-	192	70	187	261	157	82	1.58
W	This work	PBE	300	127	335	474	214	126	0.97
W	Ab initio [39]	PBE	304	156	400	527	192	149	0.89
W	Exp. [39]	-	314	163	418	533	205	163	0.99

**Table 2:** Summary of the ab initio results from this study, those reported in [42], and the experimental values obtained for pure HCP Ti at 4 K [43].

Metal	Source	Functional	C_11_ [GPa]	C_12_ [GPa]	C_33_ [GPa]	C_13_ [GPa]	C_44_ [GPa]
Ti	This work	PBE	176	84	201	74	46
Ti	Ab initio [42]	PBE	180	53	169	65	42
Ti	Exp. [43]	-	176	94	191	68	51

**Table 3:** The known isotropically elastic alloys, and the results determined by PBE.

Alloy	e/a Exp.	% at. Exp.	e/a PBE	% at. PBE	A Exp.	A PBE
TiV	4.72 [3]	Ti-72V	4.44	Ti-45V	1 [3]	1
TiNb	-	-	4.53	Ti-53Nb	-	1
TiMo	-	-	4.59 [25]	Ti-30Mo	-	0.98 [25]
W	6 [39]	W	6	W	1	0.97

## Data Availability

The original contributions presented in this study are included in the article. Further inquiries can be directed to the corresponding authors.
